# Similar Microsatellite Allelic Distribution Between *Anopheles darlingi* Population Collected by Human Landing Catch or Mosquito Magnet Traps in French Guiana

**DOI:** 10.3390/tropicalmed10060174

**Published:** 2025-06-18

**Authors:** Laetitia Ferraro, Sébastien Briolant, Mathieu Nacher, Samuel Vezenegho, Antoine Adde, Christophe Nguyen, Pascal Gaborit, Jean Issaly, Romuald Carinci, Vincent Pommier de Santi, Romain Girod, Isabelle Dusfour, Hervé Bogreau

**Affiliations:** 1 Unité d’Entomologie Médicale, Institut Pasteur de la Guyane, 97306 Cayenne, France; laetitiaferraro1@gmail.com (L.F.); vsambumuh@yahoo.com (S.V.); antoine.adde@eawag.ch (A.A.); pgaborit@pasteur-cayenne.fr (P.G.); jissaly@pasteur-cayenne.fr (J.I.); rcarinci@pasteur-cayenne.fr (R.C.); romain.girod@pasteur.fr (R.G.); idusfour@gmail.com (I.D.); 2 Unité de Parasitologie et Entomologie, Département de Microbiologie et Maladies Infectieuses, Institut de Recherche Biomédicale des Armées (IRBA), 13005 Marseille, France; christophenguyen2005@gmail.com (C.N.); hervebogreau@yahoo.fr (H.B.); 3 Unité mixte de recherche Risques Infectieux Tropicaux et Microorganismes Emergents, Aix Marseille Université, Service de santé des armées, Assistance publique-Hôpitaux de Marseille, 13005 Marseille, France; v.pommierdesanti@gmail.com; 4 Institut Hospitalo-Universitaire, Méditerranée Infection, 13005 Marseille, France; 5 Centre d’Investigation Clinique Antilles-Guyane—Inserm1424, Hôpital de Cayenne, 973006 Cayenne, France; mathieu.nacher66@gmail.com; 6 Epidémiologie des Parasitoses Tropicales, Equipe d’Accueil 3593, Université de Guyane, 97306 Cayenne, France; 7 Centre d’Epidémiologie et de Santé Publique des Armées (CESPA), 13014 Marseille, France

**Keywords:** *Anopheles darlingi*, Amazonia, population genetic structure, microsatellites, collection methods, collection time, malaria

## Abstract

*Anopheles darlingi* is a major malaria vector in South America. Understanding its population dynamics is critical for designing effective vector control strategies. While various *Anopheles* collection methods exist, they may sample distinct populations. Microsatellite genotyping across nine loci was performed to characterize *An. darlingi* populations, which were collected in French Guiana between 6:30 p.m. and 7:00 a.m. using human landing catch (HLC) or Mosquito Magnet^®^ (MM) traps. Traps were arranged in a 3 × 3 Latin square design to minimize possible effects of geographical position. Pairwise *F*_ST_ index and discriminant analyses of principal components (DAPC) were used to make comparisons. A total of 431 *An. darlingi* were analyzed. No significant genetic differentiation was observed between collection methods or time slots (*F*_ST_ values non-significant, *p* > 0.25), with DAPC revealing a single genetic cluster. Despite documented phenotypic variations, no significant population structure was detected among *An. darlingi* sampled in a rural village in French Guiana via collection methods or time slots. These findings confirm that mosquitoes collected with these various methods or time slots are suitable for the molecular studies of *An. darlingi* in French Guiana. In this context, Mosquito Magnet^®^ traps could also represent an alternative to the now controversial human landing catch.

## 1. Introduction

Malaria vector fight, control, and surveillance are increasingly relying on molecular data [1]. Monitoring insecticide resistance markers clearly enables the optimization of control strategies [2]. Studies of the genetic structure of malaria vector populations also provide a better understanding of the distribution and dynamics of these populations, which is useful for entomological surveillance [1] and for adapting interventions to control malaria. The capture of *Anopheles* mosquitoes is the essential first step in conducting genetic population studies. However, as with classical entomology, the capture methods used in such studies are not yet standardized. Additionally, the use of multiple capture methods and time slot collection approaches can introduce sampling biases (e.g., species bias [3,4]), but the hypothesis that different methods may sample different populations has never been tested. Before initiating a study of *Anopheles* genetic diversity at the scale of French Guiana, a French territory in South America, we aimed to assess whether molecular data from *Anopheles* captured by human landing catch (HLC) or Mosquito Magnet^®^ (MM) traps or during different time slots were comparable. If not, standardizing capture methods would become essential for studying vector population genetics.

*Anopheles darlingi* Root, 1926, the most abundant *Anopheles* species in Amazonian urban and forest areas [5], is a predominantly anthropophilic vector with highly aggressive feeding behavior typically active after dusk [6]. This mosquito is recognized as an important malaria vector in French Guiana [7,8]. It exhibits considerable plasticity in its biting behavior (time and location of bites, host preference) in response to local environmental conditions across the Americas [9].

Although no genetic variability study has ever been conducted in French Guiana, other studies have identified microstructures in *Anopheles* populations. A study in Acre State, Brazil, reported behavioral differences between populations from adjacent capture sites, indirectly suggesting microstructures in *An. darlingi* populations (variable biting times and exophilic/endophilic behavior were observed depending on the season) [10]. Microgeographical genetic differentiation of *An. darlingi* populations have been reported in the Western Amazon region of Brazil, from two sites 60 km apart [11] or on a smaller spatial scale near the outskirts of Porto Velho city center, linked to preferences for different breeding sites [12].

Sympatric *Anopheles* populations have also been reported, suggesting possible partial reproductive isolation within the same geographic area. In Burkina Faso, differences in swarming behavior for two species (*An. coluzzii* and *An. gambiae* s.s.) may have facilitated reproductive isolation, indicating the presence of two local sympatric populations [13]. Sympatric populations of *An. funestus* have exhibited behavioral differences in larval habitats, with some evidence of variation in adult resting behavior [14]; populations of *An. vestitipennis* in Mexico have also shown comparable behavioral divergence [15]. Such reproductive isolation in *Anopheles* populations can also be highlighted at the molecular level, as previously demonstrated in Cameroun for *An. gambiae* [16], in Sri Lanka for *An. culifacies* E [17] and in Manaus (Brazil) for *An. darlingi* [18].

Although controversial, the “gold standard” for *Anopheles* collection in the field and assessing malaria transmission risk remains the HLC, as it directly measures the human biting rate (HBR) [19]. Many other collection methods—including CDC (Center for Disease Control) Light traps, MM traps, and BG-Sentinel traps [20]—have been developed [3,21,22] to (i) avoid human contact with potentially infected mosquitoes—which raises ethical concerns—and (ii) enable the standardization of measurements by eliminating inter-individual variability in mosquito attractiveness [23]. However, the abundance of mosquitoes caught, species richness, and Simpson’s diversity index can differ between trapping methods [24]. The inter-species bias has not been linked to the sampling method used for malaria vectors [25,26].

All previously cited traps use natural, synthetic, physical, or visual attractants, with varying effectiveness across *Anopheles* species [24] or even between populations [15]. HLC relies on natural host attractiveness, where humans act as biological bait to attract and capture mosquitoes. Traps aim to replicate these baits by combining synthetic compounds with physical and visual attractants (volatile molecules called kairomones, light, color, heat, and CO_2_ emission) to mechanically and systematically collect insects with varying efficiency depending on the *Anopheles* species [20]. Given the potential presence of several parapatric or even sympatric *Anopheles* populations with distinct trophic behavior, as previously described [11,12], a selection bias can be expected depending on the collection methods used.

All comparative studies evaluating collection methods usually focus on metrics such as the number of *Anopheles* caught [21], the percentage of parous females [3,20,22], species richness [3,24], or biting behavior [27]. However, none of these studies have established whether different collection methods collect the same genetic populations. If distinct *Anopheles* populations are captured by different methods, this could distort population genetic structure analyses, particularly in studies using multiple collection approaches. Additionally, comparing results across studies employing different methods would become unreliable. Given the ethical reluctance to use HLC as the standard method for collecting anthropophilic *Anopheles*, assessing potential biases in genetic structure due to trap type and attractants is critical. To date, no published data has addressed this fundamental methodological question, which is essential for evaluating collection-related biases in genetic studies.

Given the variable periods of aggressiveness observed in different *Anopheles* species and even populations [15,25], the time of capture could also introduce selection biases in population genetic structure studies that employ multiple capture time slots. It is, therefore, essential to investigate whether different time slots attract the same *An. darlingi* populations.

In French Guiana, previous studies using the same dataset have highlighted differences in classical entomological parameters between capture methods. For instance, varying proportions of *An. darlingi* caught, and female parity rates were reported in Blondin between two sampling methods: HLC and MM traps baited either with octenol or lurex™ [3]. Differences in percentages of *An. darlingi* caught between collection time slots were also documented in Blondin [28]. We aimed to determine whether intra-species (population) sampling bias could also be detected using molecular data. To test the hypothesis that different collection methods or time slots select distinct *An. darlingi* populations, mosquitoes were collected from sentinel sites in French Guiana using two methods: HLC and MM traps baited either with octenol or lurex™. Three time slots (evening, middle of the night, and early morning) were compared. Mosquitoes were genotyped at microsatellites’ loci to assess genetic diversity and other usual parameters across sampled populations.

## 2. Materials and Methods

### 2.1. Study Site and Mosquito Collections

**Figure 1:**
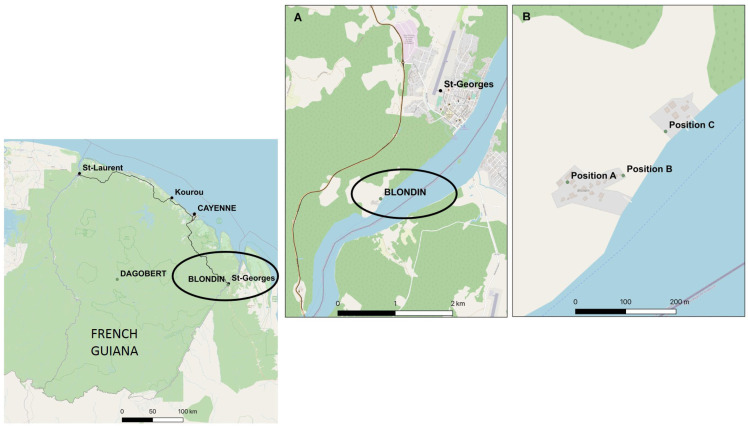
Map of collection sites of *Anopheles darlingi* in Blondin, French Guiana, between September and November 2013 sourced from openstreet map, IGN, and SIG Guyane. (**A**) Map showing Blondin, a hamlet near the town of Saint Georges de l’Oyapock. (**B**) Map showing the three *Anopheles* collection positions within the village of Blondin. GPS coordinates of each position are as follows. Position A: 3°52′32.42″ N, 51°48′54.98″ O. Position B: 3°52′32.85″ N, 51°48′51.39″ O. Position C: 3°52′35.71″ N, 51°48′48.66″ O. Distances between each trap position: 230 m between positions A and C, 111 m between positions A and B, and 146 m between positions B and C.

All mosquitoes were collected in French Guiana, a French overseas territory located in northeastern South America, where malaria transmission remains present [29]. *Anopheles darlingi*, subgenus *Nyssorhynchus*, is considered the major malaria vector in French Guiana [30]. This territory is endemic to both *Plasmodium falciparum* and *P. vivax* [31]. *Anopheles darlingi* specimens were collected in Blondin (03°52′30″ N, 51°48′54″ W) (Figure 1). Blondin is a hamlet located along the Oyapock River (at the border between French Guiana and Brazil) near the town of Saint Georges de l’Oyapock.

**Figure 2:**
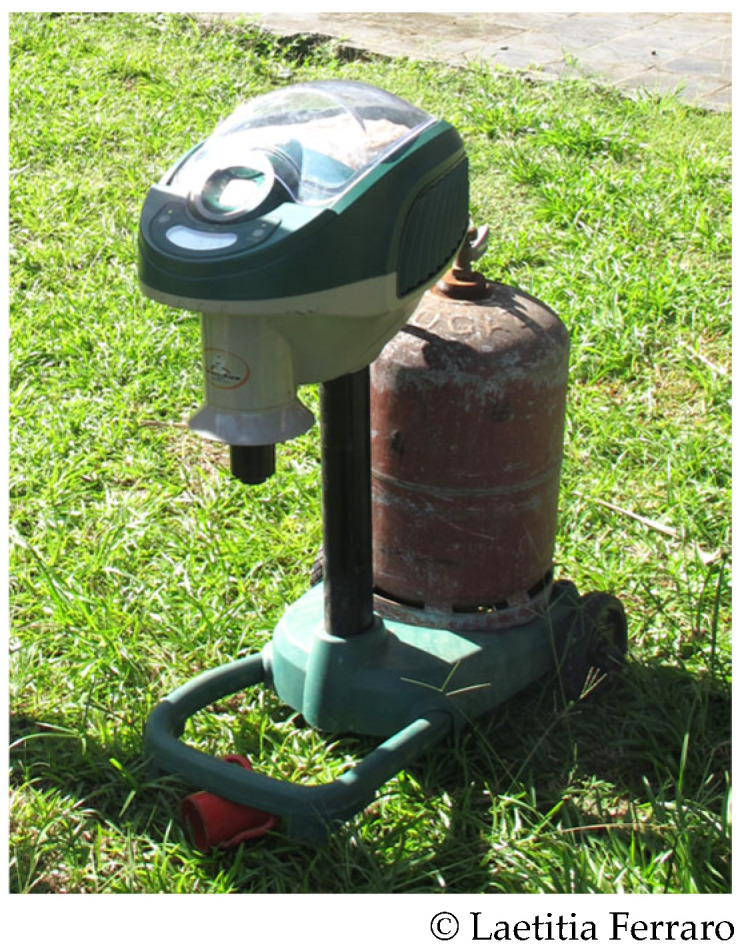
Photograph of a Mosquito Magnet^®^ trap.

To compare collection methods, adult female mosquitoes were collected outdoors in Blondin using either HLC or MM baited with octenol (that mimics human perspiring) or lurex (that mimics naturally occurring human skin scents) as a mosquito attractant (Figure 2). *An. darlingi* is predominantly anthropophilic; traps designed to preferentially capture anthropophilic species were selected to approximate the HLC method. Collections occurred between September and November 2013 (dry season) when *An. darlingi* populations in French Guiana peak [3]. Capture sessions spanned from 6:30 p.m. to 7:00 a.m. to align with the vector’s peak biting activity [28]. To mitigate potential selection biases related to trap position (e.g., proximity to breeding sites, geographical isolation, etc.) and account for the unknown vector population structure in Blondin, a 3 × 3 Latin square was implemented. Three trap positions were established (Figure 1), with each position assigned one trap type (HLC, Octenol MM, or Lurex MM). Trap types were rotated across positions during each capture session. Each session ran from 6:30 p.m. to 7:00 a.m. the following day (Table 1 and Supplementary Table S1).

Time slot comparisons were based on samples collected in Blondin, where captures were conducted continuously between 6:30 p.m. and 7:00 a.m. at each position, except for HLC, which did not operate between 10:30 p.m. and 05:00 a.m. Mosquitoes captured in MM were collected at 10:30 p.m., 05:00 p.m. and 07:00 p.m. For HLC, two humans performed HLC at each position. The time slots compared were evening (06:30 p.m.–10:30 p.m.), middle of the night (10:30 p.m.–05:00 a.m.), and early morning (05:00 a.m.–07:00 a.m.).

To avoid selection bias due to seasonal variation in the determination of *Anopheles’* genetic structure, all mosquitoes were collected over a short period (3 months) within the same season. The species composition of *Anopheles* and the parity rate of *An. darlingi* by sampling method have previously been evaluated [3], as well as the HBR, the percentage of *An. darlingi* caught, and the *An. darlingi* parity rate by time slot [28]. Total mosquito density and density/hour/capture session by trap position were also assessed (Supplementary Table S2).

To confirm that the selected microsatellite markers could differentiate between two distinct Guyanese populations, we genotyped 91 *An. darlingi* specimens from another site: Dagobert, a gold mining area located in the forest of central French Guiana. Adult female mosquitoes were collected outdoors using only the HLC method between November and December 2013 (dry season), from 7:00 a.m. to 10:30 p.m.

All mosquitoes were kept desiccated, individually placed into microtubes, and then stored frozen until laboratory analysis. Species identification was performed using morphological taxonomic keys specific to the region [32,33].

### 2.2. Extraction of DNA for Genotyping

DNA was extracted from one leg of each mosquito using the MagMax™ automated system with MagMax™-96 DNA Multi-sample kit for 96-well plate formats from Applied Biosystems^®^, Foster City, CA, USA, according to the manufacturer’s recommendations (isolation of genomic DNA from solid tissue). Samples were incubated with proteinase K at 56 °C for one hour to facilitate tissue disruption without the use of a mechanical homogenizer.

### 2.3. Microsatellite Genotyping

Nine microsatellite loci were genotyped (Supplementary Table S3). All primer pairs originated from published literature: seven were described by Conn et al. [34], and two were sourced from Angêlla et al. [12]. Primer sequences for loci ADC01, ADC28, ADC110, ADC137, ADC138 and ADMP9 were used without modification. Due to amplification failure for loci ADC02, ADC29, and ADC107, primers were redesigned with Primer Express^®^ software, version 3.01 (Applied Biosystems^®,^ Foster City, CA, USA), as detailed in Supplementary Table S3. These putative neutral microsatellite markers have not yet been mapped to *An. darlingi* chromosomes.

Each locus was amplified by multiplex Polymerase Chain Reaction (PCR) using the Type-it^®^ Microsatellite PCR kit from Qiagen^®^, Hilden, Germany, with fluorescently end-labeled (FAM™, NED™, PET™ or VIC™, Applied Biosystems^®^, Foster City, CA, USA) reverse primers. Two panels were assembled. The first mixed ADC01, ADC28, ADC29, ADC110, and ADC138, with an optimal annealing temperature of 64 °C. The second panel comprised ADC02, ADC107, ADC137, and ADMP9, with an optimal annealing temperature of 62 °C. PCR reactions were prepared with 1x of Master Mix (containing HotStarTaq^®^ Plus DNA Polymerase, Type-it Microsatellite PCR Buffer with 6 mM MgCl^2^ and dNTPs), 0.2 µM of each primer, RNase free-water to a final volume of 20 µL, and 5 µL of template DNA. Amplifications were performed on a Biometra^®^, Göttingen, Germany, T1 Thermocycler under the following conditions: 5 min at 95 °C, 28 cycles at 95 °C for 30 s, panel-specific annealing temperature for 90 s, and 72 °C for 30 s followed by a final extension at 60 °C for 45 min. For capillary electrophoresis on an ABI Prism^®^ 3130xl Genetic Analyzer (Applied Biosystems^®^, Foster City, CA, USA), a mix was prepared with 20 µL formamide, 0.5 µL GeneScan™–500 LIZ^®^ Size Standard (Applied Biosystems^®^ Foster City, CA, USA) and 1.5 µL of PCR product. Electropherograms were analyzed, and sample files were generated using GeneMapper^®^ software, version 3.7 (Applied Biosystems^®^, Foster City, CA, USA).

### 2.4. Statistical Analyses

The frequency of null alleles was tested for the nine microsatellite loci using the MICRO-CHECKER software, V2.2.3. [35]. Genetic variability of sampled vector populations was estimated based on allele frequencies at each locus and Nei’s unbiased expected heterozygosity (*He*) [36]. Hardy–Weinberg equilibrium (HWE) tests (*F*_IS_) and linkage disequilibrium were analyzed using permutation procedures in GENETIX software, V4.05.2. [37]. Population structure comparisons between *An. darlingi* groups collected via different methods and time slots were conducted using pairwise *F*_ST_ estimates [38,39]. The *p*-values after sequential Bonferroni correction were computed using FSTAT software, V2.9.4 [40]. Permutation-based statistical tests were performed with 10,000 permutations. Discriminant analysis of principal components (DAPC) [41] was performed using R software, V1.3.959 [42], with package Adegenet, version 1.3.1 [43].

## 3. Results

### 3.1. Anopheles darlingi Field Collection

A total of 3481 *An. darlingi* were collected in Blondin between September and November 2013 across the three collection method positions (Supplementary Table S2). A subsample (n = 431, Table 2) was analyzed to assess potential selection bias related to collection methods and time slots. Since the number of capture sessions varied between trap positions and collection methods (Supplementary Table S1), the total number of *An. darlingi* caught was presented by the capture session. Human Landing Catch was not conducted between 10:30 p.m. and 05:00 a.m. (middle of the night); therefore, *An. darlingi* density/hour was calculated excluding this interval, except for trap position-specific results (Supplementary Table S2). In Dagobert, 91 *An. darlingi* were caught between November and December 2013.

### 3.2. Anopheles darlingi Populations Collected with Different Methods

Microsatellite genotyping was performed on a subsample of 431 *An. darlingi* (N_HLC_ = 115, N_MM Octenol_ = 161 and N_MM-Lurex_ = 155) (Table 2). Among the nine genotyped microsatellite loci, ADC28 exhibited the lowest polymorphism (Allele number = 5 and *He* < 0.45) across all collection methods. In contrast, ADC01 was the most polymorphic (Allele number > 30 and *He* > 0.929). The results by loci are presented in Table 3 and Supplementary Table S4. Significant deviations from HWE at locus ADC138, observed in all samples (Blondin and Dagobert), were attributed to null alleles as confirmed by analysis with the MICRO-CHECKER software, version 2.2.3). This marker was excluded from subsequent population structure analyses. The unbiased genetic diversity index (*He*) revealed high diversity regardless of the collection methods (*He* _HLC_ = 0.78, *He* _MM-Octenol_ = 0.78, *He* _MM-Lurex_ = 0.77). Considering *F*_IS_, significant departures from HWE were detected at loci ADC01, ADC02, ADC28, and ADC110 after sequential Bonferroni correction (*p* < 0.002). Linkage disequilibrium tests showed a significant association for the pair ADC107–ADC137.

Global (and locus-specific) pairwise *F*_ST_ index did not reveal significant genetic differentiation between *An. darlingi* populations sampled using the three different collection methods (*F*_ST_ < 0.00146). The *p*-values range between 0.25 and 0.88 (Table 4).

**Figure 3:**
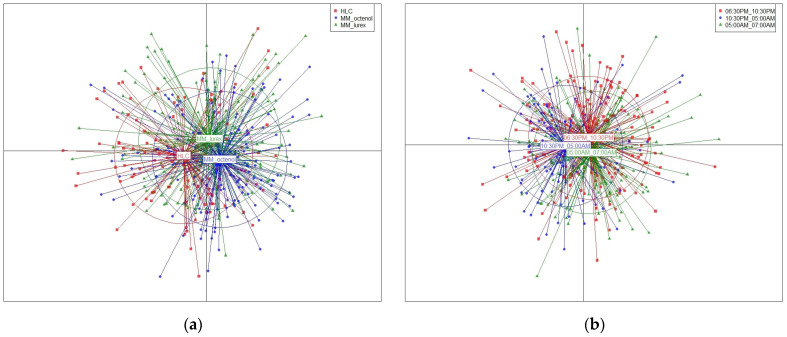
Results of clustering by DAPC of the eight-microsatellite-loci dataset of *Anopheles darlingi*. (**a**) Comparison between three capture methods with 431 mosquitoes caught in Blondin. (**b**) Comparison between three time slots at night with 431 mosquitoes caught in Blondin: evening (06:30 p.m.–10:30 p.m.); middle of the night (10:30 p.m.–05:00 a.m.); early morning (05:00 a.m.–07:00 a.m.).

Discriminant analysis of the principal component (DAPC) exhibits only one genetic population, as illustrated by three superimposed inertia ellipses (Figure 3a). Considering each trap position from the 3 × 3 Latin square, pairwise *F*_ST_ indexes between *An. darlingi* populations sampled were non-significant, regardless of the collection methods (*F*_ST_ < 0.0016 with *p*-values > 0.11667).

### 3.3. Anopheles darlingi Populations Collected at Different Time Slots

When comparing the three time slots [evening (06:30 p.m.–10:30 p.m.), middle of the night (10:30 p.m.–05:00 a.m.), and early morning (05:00 a.m.–07:00 a.m.)], the number of *An. darlingi* used for genetic comparisons was 175, 114, and 142, respectively (Table 5). Genetic diversity (*He*) across loci ranged from 0.77 (middle of the night) to 0.78 (evening). Significant departures from HWE were detected for a few samples at three loci—ADC01, ADC02, and ADC110—after sequential Bonferroni correction (*p* < 0.002) (Table 3). Linkage disequilibrium tests revealed a significant association for the pair ADC107-ADC137. Pairwise *F*_ST_ indexes between *An. darlingi* populations across time slots were below 0.00002 (Table 6). These non-significant *F*_ST_ indexes (*p* > 0.7) highlight no genetic differentiation between populations. Discriminant analysis of the principal component (DAPC) plot further confirmed this similarity, as shown by the overlapping inertia ellipses (Figure 3b). The pairwise *F*_ST_ index showed significant genetic differentiation between *An. darlingi* populations sampled in Blondin vs. Dagobert (*F*_ST_ = 0.0755, *p* = 0.05).

## 4. Discussion

Molecular biology tools are increasingly used in entomology. Their sensitivity makes it possible to address new questions. However, non-standardized sampling of *Anopheles*, using variable collection methods, involves risks of selection biases that can now be documented. The present study was designed to determine whether collection methods or trapping times could select for genetically distinct populations and bias conclusions regarding the genetic structure of exophagic *An. darlingi* populations in French Guiana.

### 4.1. Collection Methods and Sampling Bias of Anopheles darlingi

In our samples, previously published results demonstrated that the HLC method, which captured over 75% of *An. darlingi,* was more effective than MM trap baited with attractants (Octenol or Lurex) [3]. Despite these differences in *Anopheles* density, genetic diversity does not vary and remains high (*He* > 0.77) across all collection methods. This level of genetic diversity aligns with findings from prior studies using the same microsatellite markers [11,12,44]. Moreover, the differentiation index (pairwise *F*_ST_) revealed no significant genetic differences between populations collected via HLC, MM-Octenol, and MM-Lurex, as illustrated by the DAPC. This indicates that the exophagic populations of *An. darlingi* sampled by MM traps appear genetically identical to those sampled using outdoor HLC. Consequently, these two different collection methods could be used in combination to assess the genetic structure of exophagic *An. darlingi* populations in French Guiana. The methodological comparability of studies using various collection methods is thus validated. MM traps, previously identified as the best alternative to the controversial HLC [3], could be employed without introducing bias in allele frequencies at putative neutral microsatellite loci. This makes them suitable for vector population genetic studies.

This study focused exclusively on anthropophilic *An. darlingi* populations. Consequently, genetic differences such as those previously documented between *Anopheles vestitipennis* populations with differing trophic preferences were not expected [15]. However, this absence of detectable differentiation raises two nuances: (i) the sensitivity of our molecular tools and their ability to detect potential biases; (ii) the relevance of our study site in terms of encountering sympatric vector populations.

The microsatellites selected for this study were chosen based on their established ability to detect genetic structure [44,45,46,47] and even fine-scale *An. darlingi* population microstructure [12]. Moreover, our data demonstrate substantial genetic polymorphism across these markers, with an average of 17 alleles per locus (range from 7 to 38). The discriminatory power of these markers is evident, as no identical genotypes were observed in the entire sample. For instance, considering the genetic diversity (*He*) at each locus, the probability of sampling two genetically identical *An. darlingi* via HLC would be 1.4 × 10^−12^. Nevertheless, a prior study suggested that some of these markers lacked sufficient power to detect genetic microstructures in Brazilian *An. darlingi* populations [11]. By more than doubling the population sample size and incorporating an additional marker, our protocol enhances analytical power for population comparisons. Indeed, we successfully identified genetic differentiation between *An. darlingi* populations from our two study sites (Blondin and Dagobert), confirming the markers’ ability to resolve populations’ genetic differences in French Guiana.

Regarding site selection, it was impossible to determine *a priori* whether multiple *An. darlingi* populations coexisted in Blondin. However, the proximity of larval habitats, the omnipresence of forest cover, and surrounding human activities suggested significant genetic diversity could be expected (consistent with other inhabited areas of French Guiana). Observed heterozygote deficits (*F*_IS_ > 0) in Blondin populations might reflect undetected small-scale genetic heterogeneity (microstructures). A 3 × 3 Latin square design was implemented to account for this potential microstructure in analyses. However, the rare instances of linkage disequilibria likely stem from artifactual discrepancies linked to the presence of null alleles, as reported for some microsatellite markers used here [44]. The absence of significant *F*_ST_ between populations collected across Latin square positions further supports the lack of microstructural differentiation. These results align with prior studies showing no genetic difference between *An. darlingi* populations with distinct trophic behaviors (indoors vs. outdoors) [27]. The observed behavioral diversity may result from the high *An. darlingi* plasticity described [9] without causing reproductive isolation among individuals.

### 4.2. Anopheles darlingi Populations According to the Time Slots

In our samples, previously published results demonstrated higher *Anopheles* density during the evening period (6:30 p.m.–10:30 p.m.) [28]. This aligns with the following *An. darlingi* bio-ecology: In Blondin, *An. darlingi* exhibits peak biting activity between 8:30 p.m. and 10:30 p.m. or plateaus throughout the night, depending on seasonal variations [28].

Despite these density variations, no *An. darlingi* population’s genetic structure was observed between time slots. This finding aligns with a recent study [27], which is the only one, to our knowledge, that compares *An. darlingi* populations collected during different time slots (dusk vs. dawn) using genome-wide SNPs. Although the authors identified a non-random SNP distribution linked to behavior, they detected no population structure. Resolving this paradox is difficult in the absence of the fully annotated *An. darlingi* genome. Nevertheless, the authors suggest that certain markers may be associated with trophic behavior without leading to reproductive isolation.

## 5. Conclusions

As part of malaria vector control programs, entomological surveillance, including genetic population studies, requires *Anopheles* collecting at national or continental scales. Typically, multiple teams and varied laboratory protocols are involved in mosquito capture, leading to differences in trapping methods and time slots. However, molecular monitoring at these scales necessitates the comparison and aggregation of results across studies.

In this study, using microsatellite markers, we found no genetic differences between exophagic *An. darlingi* populations collected via the different methods studied (HLC, MM octenol, and MM lurex) or across time slots (evening, middle of the night, early morning) in French Guiana. *Anopheles darlingi* captured with these varied collection methods at any time can, therefore, be used in population genetic structure studies in French Guiana.

## Figures and Tables

**Table 1:** Collection methods according to the trap position and the capture session date in Blondin, French Guiana, between September and November 2013.

Capture Session Date	Trap Position			
	A	B	C	
16 and 17 September 2013		MM octenol	HLC	
17 and 18 September 2013	MM lurex	HLC		
18 and 19 September 2013	MM octenol		MM lurex	
19 and 20 September 2013	HLC			
20 and 21 September 2013		MM lurex	MM octenol	
14 and 15 October 2013	MM lurex		HLC	
15 and 16 October 2013	MM octenol	HLC	MM lurex	
16 and 17 October 2013	HLC	MM lurex	MM octenol	
11 and 12 November 2013		MM octenol	HLC	
12 and 13 November 2013		HLC		
13 and 14 November 2013	MM octenol			
14 and 15 November 2013	HLC		MM octenol	

One capture session was performed between 6:30 p.m. one day and 07:00 a.m. the next day. Collection methods were Human Landing Catch (HLC) and Mosquito Magnet^®^ Liberty Plus baited with octenol (MM octenol) or lurex (MM lurex).

**Table 2:** Characterization of the *An. darlingi,* according to the collection methods and the trap position, sampled in Blondin, French Guiana, between September and November 2013.

Collection Methods	Trap Position A	Trap Position B	Trap Position C	Total All Trap Positions
HLC	40	37	38	115 (26.7%)
MM Octenol	41	23	97	161 (37.3%)
MM Lurex	22	55	78	155 (36%)
Total all collection methods	103 (23.9%)	115 (26.7%)	213 (49.4%)	431

The number of analyzed individuals with microsatellites, with relative percentages indicated in parentheses.

**Table 3:** Genetic diversity of *An. darlingi* populations from French Guiana according to the collection methods and time slots.

			COLLECTION METHODS (N = 431)						TIME SLOTS (N = 431)					
	Allele Size (bp) N = 522	Allele Number N = 522	HLC N = 115		MM Octenol N = 161		MM Lurex N = 155		Evening N = 175		Middle of the Night N = 114		Early Morning N = 142	
			*He*	*F*_IS_	*He*	*F*_IS_	*He*	*F*_IS_	*He*	*F*_IS_	*He*	*F*_IS_	*He*	*F*_IS_
ADC01	153–263	38	0.933	0.113 *	0.941	0.102 *	0.929	0.106 *	0.935	**0.085**	0.928	0.152 *	0.935	0.094 *
ADC02	131–223	21	0.883	0.315 *	0.862	0.374 *	0.867	0.227 *	0.880	0.306 *	0.855	0.324 *	0.868	0.290 *
ADC28	125–137	7	0.450	−0.013	0.421	0.159 *	0.441	0.107	0.438	**0.122**	0.430	0.123	0.436	0.031
ADC29	246–316	19	0.829	0.036	0.838	0.023	0.844	0.008	0.835	0.009	0.849	**0.083**	0.830	−0.016
ADC107	125–201	14	0.668	**0.199**	0.714	0.086	0.697	**0.102**	0.712	**0.096**	0.694	0.077	0.677	**0.189**
ADC110	160–184	12	0.819	0.074	0.803	0.056	0.779	**0.100**	0.797	0.034	0.794	0.239 *	0.818	0.015
ADC137	121–149	13	0.791	0.024	0.771	0.001	0.747	−0.062	0.764	−0.023	0.761	0.008	0.783	−0.016
ADMP9	166–214	15	0.872	0.023	0.863	−0.015	0.875	0.042	0.872	0.043	0.859	−0.021	0.875	0.011
Total		142	0.781	**0.103**	0.776	**0.097**	0.772	**0.079**	0.779	**0.084**	0.771	**0.125**	0.778	**0.076**

Ranges of allele size, allele number, genetic diversity (*He*: expected diversity), and deviation (*F*_IS_) from the Hardy–Weinberg equilibrium based on the eight microsatellite loci. Sampling was taken from Blondin (N = 431) during the corresponding time slots: evening (06:30 p.m.–10:30 p.m.), middle of the night (10:30 P.M.–05:00 A.M.), and early morning (05:00 a.m.–07:00 a.m.). HLC: Human Landing Catch; MM octenol: MM baited with octenol; MM lurex: MM baited with lurex. * significant disequilibrium from the Hardy–Weinberg equilibrium after Bonferroni correction (*p*-value < 0.002). In bold is the presence of null alleles as estimated by MICRO-CHECKER. N: sample size.

**Table 4:** Genetic comparison of *An. darlingi* populations collected with the three collection methods.

Compared Populations	HLC	MM Octenol	MM Lurex
HLC	-	0.00146	0.00069
MM Octenol	0.35000	-	−0.00039
MM Lurex	0.25000	0.88333	-

The three collection methods compared were as follows: Human Landing Catch (HLC), MM baited with octenol (MM octenol), and MM baited with lurex (MM lurex). Pairwise *F*_ST_ (above diagonal) was based on the genotyping of eight microsatellite loci. Corresponding *p*-values (below diagonal) were implemented with 10,000 permutations using the Fstat software, version 2.9.3.2, and the significant *p*-value should be < 0.01667 with Bonferroni correction.

**Table 5:** Characterization of *An. darlingi,* according to the time slots and the trap position, sampled in Blondin, French Guiana, between September and November 2013.

Time of the Night	Trap Position A	Trap Position B	Trap Position C	Total All Trap Positions
Evening (6:30 p.m.–10:30 p.m.)	53	48	74	175 (40.6%)
Middle of the night (10:30 p.m.–05:00 a.m.)	18	28	68	114 (26.5%)
Early morning (05:00 a.m.–07:00 a.m.)	32	39	71	142 (32.9%)
Total all time slots	103 (23.9%)	115 (26.7%)	213 (49.4%)	431

The number of analyzed individuals with microsatellites, with relative percentages indicated in parentheses.

**Table 6:** Genetic comparison of *An. darlingi* populations collected during the different time slots.

Compared Populations	Evening	Middle of the Night	Early Morning
Evening	-	−0.00025	−0.00132
Middle of the night	0.70000	-	0.00002
Early morning	0.95000	0.76667	-

*Anopheles darlingi* populations were collected in Blondin during different time slots—evening (06:30 p.m.–10:30 p.m.), middle of the night (10:30 p.m.–05:00 a.m.), and early morning (05:00 a.m.–07:00 a.m.). Pairwise *F*_ST_ (above diagonal) was based on the genotyping of eight microsatellite loci. Corresponding *p*-values (below diagonal) were implemented with 10,000 permutations using the Fstat software, and the significant *p*-value should be <0.01667 with Bonferroni correction.

## Data Availability

The datasets analyzed during the current study are available from the corresponding author upon reasonable request.

## Supplementary Materials

The following supporting information can be downloaded at https://www.mdpi.com/article/10.3390/tropicalmed10060174/s1. Table S1: Total capture sessions according to the trap position and the collection method in Blondin, French Guiana, between September and November 2013; Table S2: Anopheles darlingi density presented by trap position, caught in Blondin, French Guiana, between September and December 2013; Table S3: Repeats and primer sequences of the microsatellite loci designed for An. darlingi; Table S4: Allele number obtained per locus per collection method or time slot for An. darlingi populations in Blondin and Dagobert.

## Abbreviations

The following abbreviations are used in this manuscript:

CDC	Center for Disease Control
DAPC	Discriminant analysis of principal component
HBR	Human biting rate
HLC	Human landing catch
HWE	Hardy–Weinberg equilibrium
MM	Mosquito magnet
