# Supplementary material for: Similar Microsatellite Allelic Distribution Between Anopheles darlingi Population Collected by Human Landing Catch or Mosquito Magnet Traps in French Guiana
Source: Trop Med Infect Dis. 2025 Jun 18;10(6):174. doi: 10.3390/tropicalmed10060174 (PMC12197347; doi:10.3390/tropicalmed10060174)
Supplement: Supplementary file 1 [file tropicalmed-10-00174-s001.zip › tropicalmed-3564044-supplementary.pdf]

Table S1. Total capture sessions according to the trap position and the collection method in Blondin, French Guiana, between September and November 2013.

		Trap position			Total capture sessions
		A	B	C	
Collection method	HLC	3	3	3	**9 (39.1%)**
	MM Octenol	3	2	3	**8 (34.8%)**
	MM Lurex	2	2	2	**6 (26.1%)**
Total capture sessions		**8 (34.8%)**	**7 (30.4%)**	**8 (34.8%)**	**23**

Table S2. *Anopheles darlingi* density presented by trap position, caught in Blondin, French Guiana, between September and November 2013.

		Total density for all capture sessions	Density per hour and per capture session
Trap position	position A	832 (23.9%)	8.1 (22.9%)
	position B	967 (27.8%)	9.9 (28.1%)
	position C	1682 (48.3%)	17.2 (48.9%)

Relative percentages are indicated in parenthesis, for HLC, the density was calculated per human (two humans make HLC by session).

Table S3. Repeats and primer sequences of the microsatellite loci designed for *An. darlingi*.

**Locus (GenBank)**	**Repeat numbers**	**Primer sequences (5′ – 3′)**	**Ta (°C)**	**Fluorochrome**
PANEL A				
ADC01[‡] (AF322185)	GA	f - GCTCTAATGATGCTCGTAACCGCT<br>r - CGACACCGCACTTGCTACTACCTG	64	FAM
ADC28[‡] (AF322187)	GA	f- CTCGTCGTCAGCGTCGTGC<br>r- TGCCCATCCACTGCGTAACGG	64	PET
ADC29[‡¤] (AF322188)	GA	f- CATGGAACGCACTGGGATTATT<br>r- CATCGCGTAATCCGAAATTGAA	64	PET
ADC110[‡] (AF322190)	GT	f- CCGAACAACAGCCAACAGCTGTG<br>r- CGTTCGACACAATCGTTACACACG	64	VIC
ADC138[‡] (AF322192)	AC	f- CTTTGAGCCGGTGCTGTGCTGC<br>r- CCATTCTCGCAGCCTCCAGGAC	64	NED
PANEL B				
ADC02[‡¤] (AF322186)	GA	f- CACACTGGGGCATCATTCATTTC<br>r- CCGGGCTATCTATTCTTTCCCACT	62	PET
ADC107[*¤] (AF322189)	AC	f- GTCCACTCCCAGGCACAC<br>r- AGCAATCGAGGCAAACTTTC	62	FAM
ADC137[‡] (AF322191)	GT	f- TCTTACGGGAATGGTGCGACGCTC<br>r- CAGCCACCCATACGCTGTTGACCA	62	NED
ADMP9* (DV729762)	AAC	f- ACAACGTCCAATGCAACAAC<br>r- CTCGAGGGCTTTCTGTATCG	62	VIC

Ta: annealing temperature use in this study, the GenBank accession number is listed below the corresponding locus,
‡ described by Conn *et al.*(J. E. Conn et al. 2001),
* described by Angêlla *et al.*(Angêlla et al. 2014),
¤ Redesigned for this study

Table S4. Allele number obtained per locus per collection method or time slot for *An. darlingi* populations in Blondin and Dagobert.

	Blondin						Dagobert
**Locus**	HLC	MM Octenol	MM Lurex	Evening	Middle of the night	Early morning	
ADC01	30	36	31	32	33	35	17
ADC02	20	18	16	20	15	18	13
ADC28	5	5	5	5	5	5	4
ADC29	16	18	17	18	18	16	14
ADC107	6	9	9	10	8	7	5
ADC110	12	11	10	11	11	11	9
ADC137	10	10	9	10	10	10	10
ADC138	10	10	8	9	9	10	7
ADMP9	12	12	15	15	12	13	9
Totality of loci	121	129	120	130	121	125	88

Sample size: Blondin: N = 431, Dagobert: N=91, corresponding time slot: evening (06:30 PM – 10:30 PM), middle of the night (10:30 PM – 05:00 AM) and early morning (05:00 AM – 07:00 AM).
